# Interventions to Improve Uptake of Direct-Acting Antivirals for Hepatitis C Virus in Priority Populations: A Systematic Review

**DOI:** 10.3389/fpubh.2022.877585

**Published:** 2022-06-24

**Authors:** David Ortiz-Paredes, Afia Amoako, Taline Ekmekjian, Kim Engler, Bertrand Lebouché, Marina B. Klein

**Affiliations:** ^1^Center for Outcome Research and Evaluation, Research Institute of the McGill University Health Center, Montreal, QC, Canada; ^2^Department of Epidemiology, Biostatistics, and Occupational Health, McGill University, Montreal, QC, Canada; ^3^Medical Libraries, McGill University Health Center, Montreal, QC, Canada; ^4^Department of Family Medicine, McGill University, Montreal, QC, Canada; ^5^Division of Infectious Diseases/Chronic Viral Illness Service, Department of Medicine, Glen site, McGill University Health Center, Montreal, QC, Canada; ^6^CIHR Canadian HIV Trials Network, Vancouver, BC, Canada

**Keywords:** Hepatitis C, antiviral agents, people who inject drugs, Indigenous peoples, sexual and gender minorities

## Abstract

**Background & Objective:**

Access to Hepatitis C (HCV) care remains suboptimal. This systematic review sought to identify existing interventions designed to improve direct-acting antiviral (DAA) uptake among HCV infected women, people who inject drugs (PWID), men who have sex with men (MSM), and Indigenous peoples.

**Methods:**

Studies published in high- and middle-income countries were retrieved from eight electronic databases and gray literature (e.g., articles, research reports, theses, abstracts) were screened by two independent reviewers. Identified interventions were summarized using textual narrative synthesis.

**Results:**

After screening 3,139 records, 39 studies were included (11 controlled comparative studies; 36 from high-income countries). Three groups of interventions were identified: interventions involving patients; providers; or the healthcare system. Interventions directed to patients included care co-ordination, accelerated DAA initiation, and patient education. Interventions involving providers included provider education, telemedicine, multidisciplinary teams, and general practitioner-led care. System-based interventions comprised DAA universal access policies and offering HCV services in four settings (primary care, secondary care, tertiary care, and community settings). Most studies (30/39) described complex interventions, i.e., those with two or more strategies combined. Most interventions (37/39) were tailored to, or studied among, PWID. Only one study described an intervention that was aimed at women.

**Conclusions:**

Combining multiple interventions is a common approach for supporting DAA initiation. Three main research gaps were identified, specifically, a lack of: (1) controlled trials estimating the individual or combined effects of interventions on DAA uptake; (2) studies in middle-income countries; and (3) interventions tailored to women, MSM, and Indigenous people.

## Introduction

Hepatitis C virus (HCV) is a global health problem affecting around 58 million people worldwide (1). It is estimated that 7.2 million people will die from this disease between 2015 and 2030 (2). The introduction of direct-acting antivirals (DAAs) in 2013 has made HCV a curable chronic viral infection. DAAs have shown cure rates in real-world settings of over 95% with an 8- to-12-week once daily treatment with few or no side effects (3–5).

Hepatitis C virus elimination is now a possibility, which led the World Health Organization (WHO) to propose a global strategy aimed at eliminating HCV as a public health threat by 2030 (2). The current approach to achieving this goal is to target efforts to priority populations among whom HCV prevalence and incidence are high (6). Examples of these populations are people who inject drugs (PWID), men who have sex with men (MSM), and Indigenous people (3).

Each priority population has its own individual characteristics and faces distinct challenges, which impact its ability to access DAAs. PWID–especially female PWID–have reported stigma and discrimination, including in healthcare settings (7, 8). Homelessness, limited geographical access to HCV care, and criminalization of injection drug use (IDU) are other obstacles identified among PWID that could hinder their HCV treatment access (7). For MSM, unprotected anal intercourse, group sex, and the use of drugs during sex are sexual behaviors that have been associated with an increased risk of HCV infection (9–11). The incidence of HCV infection and re-infection remain particularly high among human immunodeficiency virus (HIV)-positive MSM (11–13). In Canada, Indigenous people experience five to 11 times higher HCV infection rates when compared to non-Indigenous people (12, 14). IDU, HCV-related stigma, and the impacts of colonization and intergenerational trauma are some of the main HCV treatment barriers for Indigenous peoples (12, 14–16). Being HCV-positive has been associated with greater mortality among Indigenous people especially among women (17–19).

There is a call for developing strategies tailored to key populations' needs (6, 7). Improving DAA uptake is increasingly seen as the critical step in the HCV care cascade, as a decrease in treatment initiation rates might prevent countries like Canada from achieving the WHO targets by 2030 (20). Previous systematic reviews have been published elsewhere. However, some include pre-DAA interventions (21) and others have focused on PWIDs or prisoners only (21, 22). The purpose of this systematic review was to map DAA-era interventions aimed at improving HCV uptake among a broad range of priority populations (e.g., PWID, MSM, Indigenous peoples and women). We focused on high- and middle-income countries to parallel the healthcare setting in Canada, as this review aims to inform HCV elimination efforts in this country.

## Methods

This systematic review was conducted and reported following the Preferred Reporting Items for Systematic Reviews and Meta-Analyses (PRISMA) statement (23). The protocol was registered on PROSPERO (CRD42020158607).

### Search Strategy

A comprehensive literature search was initially run on December 5 2019 and then rerun on February 10 2021.The following databases were searched for relevant studies: Medline (*via* Ovid 1946 to February 09, 2021); The Cochrane CENTRAL Register of Controlled Trials & Cochrane Database of Systematic Reviews (*via* Wiley, Issue 2 of 12, February 2021); Embase (*via* Ovid 1974 to 2021 February 09), CINAHL (*via* Ebsco), Biosis (*via* Clarivate Analytics), Global Health (*via* Ovid 1973 to 2021 Week 05), Global Index Medicus and Scopus.

The search strategy was inductively designed in close collaboration with a librarian (author TE) and used text words and relevant Medline indexing terms to identify studies on DAA initiation interventions in PWID, MSM, Indigenous people, and women (see Supplementary Data 1). Search terms were identified from the following four key concepts: (1) HCV; (2) DAA access and uptake; (3) populations of interest; and (4) high- and middle-income countries. Preliminary search strategies were run and the resulting strategy underwent a peer review process by a second librarian. The Medline strategy was applied to all databases, with modifications to search terms as necessary. No language limits were applied during the search. The results were limited to studies published after 2013, when DAAs became more widely available. Search strategies were peer-reviewed by a second librarian. Clinical trial registries were also searched (clinicaltrials.gov; International Clinical Trials Registry Platform) for relevant research in progress. Gray literature (e.g., articles, research reports, theses, abstracts) was retrieved from databases, organizational and governmental websites, and conference websites (see Supplementary Data 2). Further studies were identified in Web of Science and Scopus on May 8, 2020, by carrying out citation searches for the reference lists of the so far included studies. Duplicates were removed with the EndNote software's duplicate checker, using various field combinations, followed by a manual screening.

### Eligibility Criteria

Primary investigations reported in either manuscripts or conference material were included if they described or evaluated interventions (e.g., programs, initiatives, models of care) to address HCV treatment initiation, uptake or access; they were conducted in middle-lower, middle-upper- or high-income countries according to the 2019 World Economic Situation and Prospects classification (24); interventions were targeted to, and study participants belonged to, the populations of interest (PWID, MSM, Indigenous peoples, women); they presented information on DAA initiation rates; they were written in English; and if they were published after 2013 until present.

Manuscripts or conference material were excluded when they focused on HCV treatment other than DAAs; they were conducted in lower-income countries according to the 2019 World Economic Situation and Prospects classification (24); their participants were not part of the population of interest; they did not describe interventions; they were qualitative studies; and when they were not primary research (e.g., editorials, commentaries, essays, letters to the editor, reviews).

The inclusion of unpublished conference material in systematic reviews has advantages and disadvantages. Critical appraisal of abstracts could be challenging, and they often report preliminary results. Nevertheless, abstract inclusion may increase comprehensiveness, timeliness of information and precision, while decreasing publication bias (25, 26). We decided to include conference proceedings material as long as it reported complete results (i.e., protocols and research in progress were excluded). We judged this decision would improve the breadth of our results as not including conference material could potentially omit interventions.

### Studies Selection

Records identified were imported into Rayyan QCRI (27), which is an online tool that facilitates collaboration and the screening process during systematic reviews. Titles and abstracts were screened by two independent reviewers (authors DOP, AA) using the eligibility criteria described above; conflicts were resolved through consensus-based discussion. Then, the same selection process was used for the full texts of selected records.

### Data Extraction

In addition to all information related to the intervention(s) under investigation, the data extracted comprised: country, study design, targeted population (PWID, MSM, Indigenous, women), and data on DAA initiation rates. This information was organized using a data extraction table. The authors were contacted *via* email when clarification was needed.

### Evidence Synthesis

The data extraction table served to inductively identify homogeneous clusters of existing interventions. This information was synthesized using textual narrative synthesis (28). This process was initially conducted by author DOP using NVivo Mac 12. The initial analysis underwent a review by the co-authors, which allowed the validation and refinement of results. Due to an important heterogeneity across studies (in terms of research designs and outcomes) and the fact that most interventions were studied in conjunction with other initiatives, no meta-analyses or other quantitative synthesis methods were employed. It was not possible to estimate the effect that each individual intervention had on DAA initiation rates. Data supporting this review's results are available from the corresponding author MBK upon request.

### Critical Appraisal

The methodological quality of the included manuscripts was assessed using the 2018 version of the Mixed Methods Appraisal Tool (MMAT) (29). The MMAT is a content-validated tool that assesses the quality of a wide variety of methodologies, including randomized controlled trials, non-randomized studies (such as cohort studies and case-control studies), and quantitative descriptive research (such as surveys and case series) (29). Thus, the MMAT was chosen as appraisal tool as it is a comprehensive tool that allows the evaluation of the most common types of empirical investigations according to their own methodological properties. Conference materials were assessed using the STROBE checklist (30), which includes 11 items.

## Results

### Study Selection

**Figure 1:**
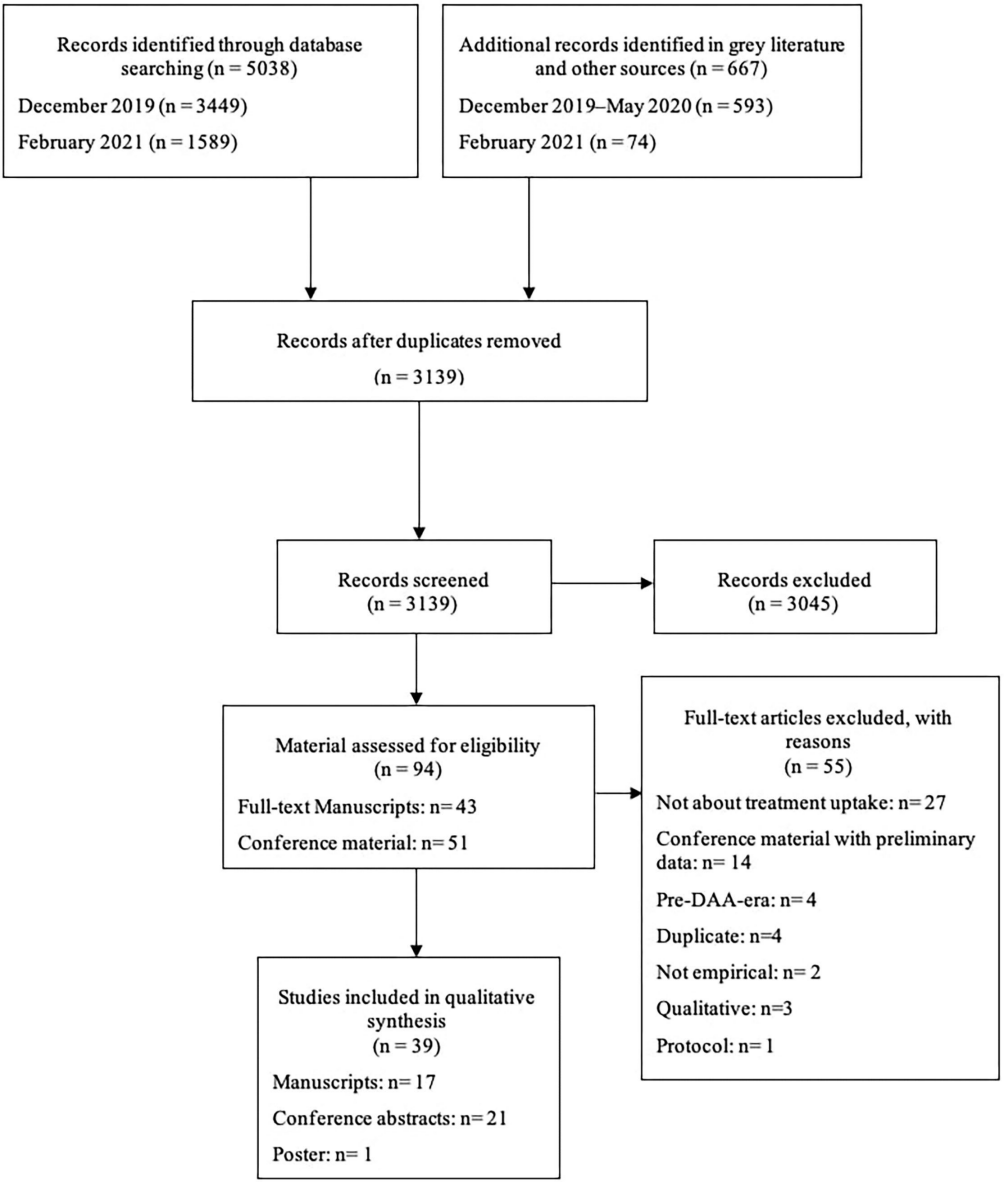
PRISMA flow diagram.

The database searches yielded 3,449 records, whereas 593 titles/abstracts were retrieved from gray literature and citation searches. After removing duplicates (*n* = 1,563) and adding the records identified during the update of the review (*n* = 660), a total of 3,139 records were reviewed and 3,045 of them were excluded following title and abstract screening. This process left a total of 94 records for full-text review (conference proceedings *n* = 51; manuscripts *n* = 43). Fifty-five records were then excluded leading to the inclusion of 39 studies (17 full-text manuscripts, 22 conference publications) (see Figure 1).

### Study Characteristics

The characteristics of selected studies and a summary of their findings can be found in Supplementary Table 1. Most studies (92%; 36/39) were conducted in high-income countries. One study (31) took place in an upper-middle income country, whereas two (32, 33) were led in a lower-middle-income country.

The majority of the research designs were prospective cohort studies (*n* = 24). The remaining were non-randomized trials (*n* = 3), comparative cohorts (*n* = 3), retrospective cohorts (*n* = 2), historical comparative cohorts (*n* = 2), randomized controlled trials (*n* = 2), a cluster randomized trial, a quasi-experimental trial, and a cost-effectiveness study. Only 11 studies had a control arm.

### Critical Appraisal

Supplementary Table 1 also contains the number of criteria that each study met out of the possible five using the MMAT (29) and the possible 11 using the STROBE. Ward et al. (34) and Harrison et al. (35) are publications of the same study and were assessed as a set, as recommended when using the MMAT (36). Fifteen manuscripts were appraised as non-randomized studies, with Coopet et al. (37), Falade-Nwulia et al. (38), and Harrison et al. (35) being the most methodically robust. Other reasons why studies scored lower included not accounting for confounders and the possibility of unplanned co-interventions or contamination. Wade et al. (39) and Radley et al. (40) were the only randomized controlled trial. These trials were not blinded because of the nature of their intervention and had considerable loss to follow-up. Regarding conference abstracts, not reporting participants' inclusion/exclusion criteria and statistical methods were the two most frequently missed items from the STROBE checklist.

### Results of the Synthesis

Three clusters of interventions designed to improve DAA initiation were identified, depending on the stakeholder group they were addressing: those directed toward patients, health providers or the healthcare system as a whole (see Table 1). These interventions are explained in greater detail below.

**Table 1:** Existing interventions to improve DAA initiation among women and priority populations of PWID, MSM, and Indigenous people and number of studies reporting each intervention in brackets.

Interventions targeting patients	Care co-ordination (15)	Streamlined referrals (8)	To usual care (2)
			To community (1)
	Accelerated DAA initiation and on-site distribution (8)		
	Patient education (11)	Peer-based support (5)	
Interventions targeting providers	Provider education (5)		
	Telemedicine (3)		
	Multidisciplinary teams (15)	Nurse-led care (4)	
		Pharmacist-led care (1)	
	Specialist-supported general practitioner-led care (2)		
Interventions targeting the health system	DAA universal access (6)		
	HCV care setting (27)	Primary care-based models (5)	
		Secondary care (1)	
		Tertiary care (1)	
		Colocation in community setting (21)	Community centers (5)
			OST centers (8)
			Needle exchange services (7)
			Mobile clinics (3)

*DAA, direct-acting antivirals; MSM, men who have sex with men; OST, opioid substitution therapy; PWID, people who inject drugs*.

#### Interventions Targeting Patients

The first group concerns interventions that aim to improve patient access to, and use of, healthcare system services, as well as their HCV-related knowledge. Three core patient interventions were described across studies: care co-ordination, accelerated DAA initiation, and patient education.

Care co-ordination interventions were the most studied patient-targeted intervention (*n*=15; see Table 1). Care co-ordination referred to accompaniment of patients and the organization of individualized action plans to overcome access barriers to complete HCV care cascade steps. Care co-ordination comprises of case management and patient navigation. Care co-ordination could be given by nurses (34, 35, 41, 42), social workers (33) or harm reduction staff (43).

Under the umbrella of care co-ordination, some studies implemented streamlined referrals (31, 33–35, 38, 44–46). This intervention was well-defined by Harrison et al. (35) as “streamlining and simplification of referral pathways, including immediate arrangement of clinic appointments taking into account client preferences for timing and integration of HCV appointments with their commitments to opiate substitution therapy.” Through this intervention, patients could be referred to either usual care (47) or community care (e.g., with general practitioners or community nurses) (48).

Patients' access to treatment was also supported with accelerated DAA initiation and on-site distribution. Martel-Laferriere et al. (49) describe an accelerated model of care at an addiction clinic in Canada which involved performing a single-day HCV assessment (including medical assessment, HCV viral load, and transient elastography), which concluded by the determination of DAA eligibility and on-site DAA initiation at a second visit. Distribution of DAAs was also implemented in mobile clinics (50, 51), needle exchange centers (45, 52), opioid substitution therapy centers (53), pharmacies (40), and community centers (54).

Patient education regarding HCV care, prevention of reinfection, substance use, mental health, and counseling was also reported (33, 41–43, 45, 46, 53–55). Education was not supplied exclusively by healthcare providers. Indeed, all peer-based interventions included the transfer of HCV-related knowledge between patients. Three peer-based interventions were identified: taking advantage of existing social connections to engage PWID in HCV care (38), peer support (35), and group hboxmedical visits (46).

#### Interventions Targeting Healthcare Providers

The second group of interventions were targeted at providers and addressed their knowledge about HCV and the ways in which they communicate and collaborate with other stakeholders. Four distinct provider-based interventions were identified: provider education, telemedicine, multidisciplinary teams, and general practitioner-led HCV care.

Hepatitis C virus education was offered to a wide variety of professionals including primary care physicians (56, 57) and staff members who have contact with people living with HCV (34, 35, 58). Staff education sought to improve not only their HCV-related knowledge but also their communication skills when engaging patients (35). Education sessions could be led by nurses, social workers, or specialists (57, 58).

Telemedicine interventions were implemented and studied in three studies (37, 51, 53). This intervention facilitated communication between providers (37, 53), as well as patient-provider interactions. Wungjiranirun et al. (51) and Talal et al. (53) described the use of telemedicine in a mobile clinic and a drug dependence center, respectively.

The implementation of multidisciplinary teams was the most common provider intervention evaluated (*n* = 15; see Table 1). This intervention fostered interprofessional collaboration between different stakeholders (e.g., general practitioners, specialists, nurses, social workers, pharmacists). Several teams were led by nurses, who were in charge of screening, care co-ordination, referrals, patient education and patient evaluations (39, 42, 48, 58), while primary care physicians or HCV care specialists were in charge of prescribing DAAs (39, 57, 58). Other teams are pharmacist-led. For instance, Radley et al. (40) describe an intervention in which pharmacists command HCV diagnosis and treatment initiation, aided by close collaboration with nurses (for phlebotomy) and specialists (for advice in complex cases). HCV multidisciplinary teams studied as singular interventions showed a DAA initiation rate of between 24% (59) and 51% (60) among HCV positive participants. Mohsen (57) found that DAA initiation in a multidisciplinary team, which included provider education, was almost the same as in a tertiary care clinic (78% vs. 81%, respectively).

The final intervention targeting healthcare providers is general practitioner-led care. This strategy refers to a model in which primary care physicians are in charge of DAA prescription and receive support from HCV specialists (37, 56, 61).

#### Interventions Targeting the Healthcare System

The third group comprised system-based interventions and approaches to the allocation of resources and services for HCV care. These interventions included a single health policy change and offering HCV services in different settings.

Six studies discussed universal access to DAAs (62–67). DAA scale-up was achieved by making them accessible regardless of liver fibrosis stage (63, 67) or by having a government-funded unrestricted DAA access program, which made DAAs available irrespective of treatment history or drug use status (64, 65). Gottfredsson et al. (62) and Chromy et al. (66), as abstracts, do not explain how treatment scale-up was achieved. DAA universal access was found to increase DAA initiation rates by 1.8 times (95% CI, 1.4–2.4) following removal of fibrosis restrictions (63), reaching uptake rates between 41% (66) and 94% (62). This positive effect seems to be stronger the year following the introduction of DAA universal access, after which DAA uptake plateaued or decreased (63, 65). Absence of a universal DAA policy negatively affected the impact of three complex interventions in which only between 27% and 43% of participants initiated HCV treatment (37, 41, 68). DAA universal access was studied in Australia, Canada, Greece, and Iceland.

Healthcare system interventions also involved offering HCV care in four different settings: primary care, secondary care, tertiary care and community settings (see Table 1). HCV diagnosis and treatment services as well as other patient and provider interventions were implemented in primary care settings. For instance, a nurse-led multidisciplinary team implemented at a primary care setting was associated with 75% DAA initiation vs. 34% in the control group (RR = 2.48; 95% CI 1.54–3.95) (39). A total of five studies described strategies in primary care (37, 39, 46, 56, 61). The only intervention in secondary care was the implementation of an open access specialist clinic, which allowed for specialist care without the need for primary care physician referral (69). This intervention was studied on a cluster randomized trial and DAA uptake was found to be 38% compared to 6% in the control group (69). Freeman et al. (48) studied a tertiary care intervention in which hospitalized high-risk patients were identified through the screening of electronic medical records to offer inpatient HCV testing and DAA initiation upon discharge.

The final subgroup of system-based interventions involved offering services (e.g., screening, patient education, treatment initiation, adherence support, referrals) in a community setting. For instance, an intervention including colocation of care in the community, provider education, care co-ordination, streamlined referrals, and peer support was found to increase DAA uptake by 13% (95%CI 9–16) (35); Community setting strategies were the most studied system-based intervention (*n* = 21; see Table 1 and Supplementary Table 2). This approach was achieved either by collocating HCV care within other community services or through mobile clinics. HCV services were offered at community centers (31, 54), community pharmacies (40), drug dependence centers (31, 34, 35, 41, 52, 53, 55, 68), homeless facilities (31, 58), and needle exchange services (42, 43, 45, 47, 52, 68, 70). In addition, Filippovych et al. (32), Wungjranirun et al. (51), and Buchaman et al. (70) studied the implementation of mobile clinics to address barriers to HCV care.

#### Complex Interventions

**Figure 2:**
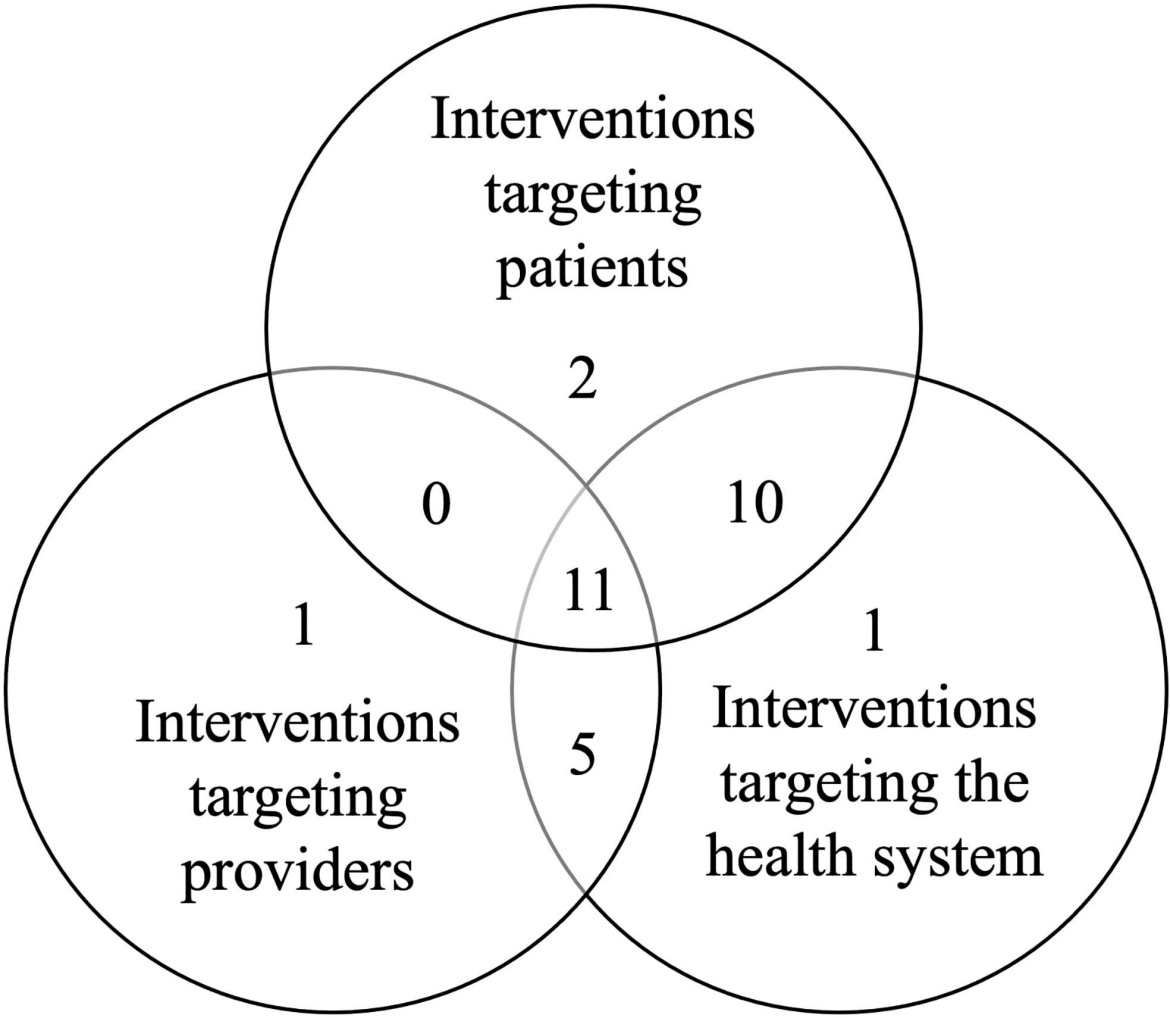
Stakeholder group(s) addressed by the 30 complex interventions.

Most studies (77%; 30/39) described *complex interventions*, which refer to strategies that combined two or more interventions. Figure 2 displays the stakeholder group (i.e., patients, health providers, health system) toward which these 30 studies directed their complex interventions. Strategies directed to the three stakeholder groups were combined in 11 studies, whereas 10 studies combined interventions that targeted patients and the health system. Interventions aimed to providers and the healthcare system were integrated in five studies. Four studies combined two or more interventions that were targeted to a single group. None of the studies described complex interventions that simultaneously targeted patients and providers, without also involving a system-based intervention. Supplementary Table 2 shows in greater detail how specific interventions were combined. DAA universal access, multidisciplinary teams, and secondary care specialist clinics were the only interventions that were not studied in conjunction with other interventions.

#### Populations for Which Interventions Were Studied

**Figure 3:**
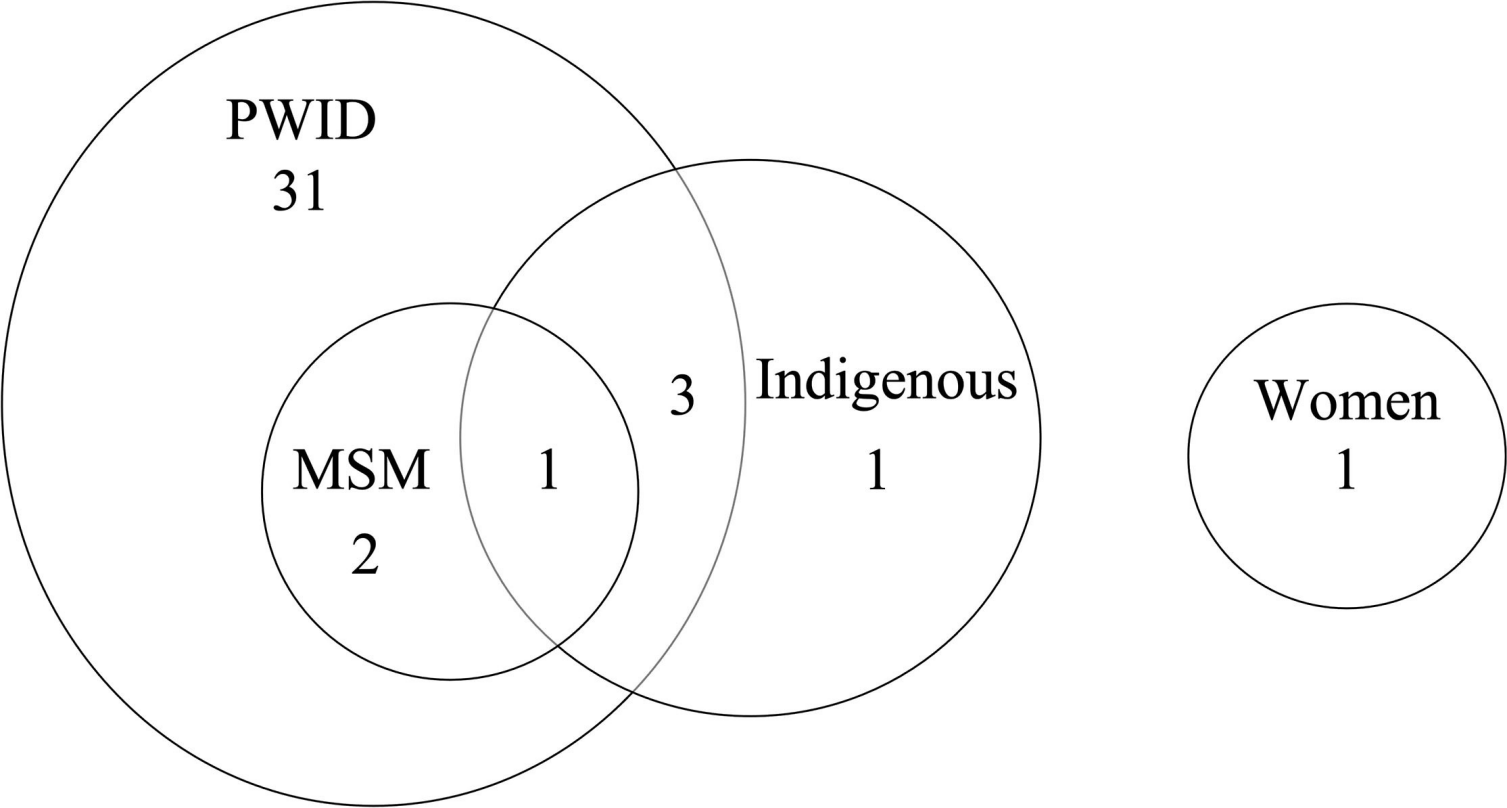
Number of studies that addressed the populations of interest. MSM, men who have sex with men; PWID, people who inject drugs.

There was an overlap in the populations studied across included studies (see Figure 3). In total, an important majority of studies (95%; 37/39) investigated their interventions in PWID, followed by Indigenous groups (*n* = 5), and MSM (*n* = 3). Only one study (54) described a strategy aimed specifically at women. The study by Saeed et al. (63)–which examined the impact of universal access to DAAs–was the only one to address the effects of this intervention simultaneously on PWID, MSM, and Indigenous patients. Martinello et al. (65) and Awali et al. (60) were the only two papers to consider both PWID and MSM. Similarly, three studies considered both PWID and Indigenous patients (43, 44, 57) (see Figure 3).

## Discussion

Instead of creating population-wide interventions, developing population-specific approaches to improve HCV care access has been proposed as the path to eliminate this chronic viral infection (6). The present systematic review examined a wide variety of databases, sources and types of studies to identify existing interventions or strategies designed to improve HCV treatment initiation among women, PWID, MSM, and Indigenous peoples focusing on high- and middle-income countries.

We identified interventions in the current literature and organized them depending on their targeted stakeholder group (see Table 1). The most commonly implemented interventions were care co-ordination for patients, multidisciplinary teams for healthcare providers, and colocation of HCV care in community settings for the healthcare system. These interventions address a wide variety of obstacles faced by all subpopulations alike, such as stigma, gaps in the continuity of care, limited geographical access and homelessness (7, 71). Our results also reflect that there is an interest in offering decentralized HCV care by placing services as close as possible to HCV patients. This indicates that HCV care is taking a similar direction as in other infectious diseases, like HIV (72) and tuberculosis (73), in which decentralized care has proven to be an effective way to engage key populations.

The majority of studies described strategies that combined more than one intervention, suggesting that complex interventions are a common approach to promote and support HCV treatment initiation in priority populations. However, since the included studies were not designed to test the effectiveness of individual components, it is unclear if there is an additive or synergistic effect of implementing all components of these complex interventions compared to simpler individual strategies.

Few studies had control groups. Most of the uncontrolled studies reported a DAA uptake rate that was greater than for the general population, estimated to be below 20% (19, 74–79); however, their interventions were implemented in heterogeneous ways and are being evaluated without robust research designs. As a consequence, the integration of these studies' findings, for instance in meta-analyses, is difficult and may create uncertainty about which strategies to prioritize. More controlled studies are thus needed when evaluating interventions to improve DAA initiation. Such results would be critical to inform modeling and selection of strategies for countries to implement HCV elimination programs.

Direct-acting antiviral universal access appeared to be another fundamental intervention. Indeed, some interventions' impact on HCV treatment uptake was negatively affected by the absence of a universal DAA access policy. However, this system-based intervention seems to have a limited impact over time. Although removing fibrosis restrictions for DAAs appears to be an important step to improve HCV treatment initiation, HCV treatment uptake decreases 1 year following DAA universal access (19, 65). Therefore, additional interventions are needed to maintain high HCV treatment uptake rates after DAA universal access is implemented. A combination of interventions targeted to patients, healthcare providers, and the allocation of HCV services is likely to be an effective strategy to maintain DAA universal access impact over time.

Our systematic review focused on identifying interventions to improve DAA uptake, as this HCV care cascade step is critical in achieving the WHO HCV elimination targets (20). However, achieving such targets also requires an efficient system capable of identifying people living with HCV. Thus, all interventions identified in the present review need to be implemented in the context of strong HCV screening programs. Other systematic reviews have identified HCV screening interventions, such as practitioner-led testing, prioritizing testing in primary care and community settings, and provider education (80, 81), all of which can be implemented in conjunction with the interventions identified in the present review.

This review indicated that most scientific research on HCV interventions for increasing DAA uptake is concentrated in high-income countries. More investigation in this area is needed in middle-income countries. Finally, most research on interventions to improve DAA initiation is conducted with PWID. While it is true that PWID are at high risk of HCV infection (82), in practice, priority populations overlap (e.g., an Indigenous patient may inject drugs) and may experience “intersectional stigma” (83). Therefore, further interventions should specifically address and be tailored to other populations, such as women, MSM, and Indigenous peoples.

This systematic review has limitations. One concerns the screening process, specifically, the exclusions of abstracts that did not explicitly mention the populations of interest. As a consequence, we may have missed some potentially relevant studies. For instance, interventions not specifically targeted at the populations of interest or those studied at a general populational level were excluded. However, this was a choice that allowed us to successfully answer our review question and still provide a comprehensive portrait of interventions aimed at improving HCV initiation among a broad range of priority populations. Another limitation was the inability to retrieve the full text of conference abstracts despite contacting their authors, which represent 56% (22/39) of selected studies. Hence, the description of many interventions was restricted by the word limits of conference material. Nevertheless, including conference abstracts improved the comprehensiveness and precision of this knowledge synthesis (25). Finally, the inclusion of articles written only in English is a limitation, as some interventions may not have been captured.

In conclusion, the identified interventions were directed to three types of stakeholders: patients, care providers, and the healthcare system. Universal access to DAAs appears to be a critical first step but is not sufficient to maintain uptake. The combination of two or more interventions appears to be a common approach to promote DAA initiation. While some interventions seem promising, estimated effectiveness was quite variable and methodologic limitations prevented conclusions about what strategies might be most valuable to scale up. Specifically, we identified three research gaps, a lack of: (1) controlled studies estimating the individual or combined effects of interventions on DAA treatment initiation rates; (2) studies in middle-income countries; and (3) investigation of interventions tailored to women, MSM, and Indigenous people. This systematic review provides a portrait of existing strategies to support DAA uptake. We hope its findings will provide a roadmap for stakeholders (e.g., patient partners, researchers, clinicians, quality improvement agents, and policymakers) to design, study, report and implement new interventions to ultimately meet WHO goals to eliminate HCV infection.

## Data Availability Statement

The original contributions presented in the study are included in the article/Supplementary Material, further inquiries can be directed to the corresponding author/s.

## Author Contributions

MK obtained the funding for the present work. MK and BL conceived the current review. TE designed the search strategy and provided substantial methodological support. DO-P and AA performed the studies selection, data extraction, and evidence synthesis. DO-P drafted the manuscript. AA, TE, KE, BL, and MK reviewed the manuscript, provided feedback, and approved the final version. MK supervised the present work. All authors have participated sufficiently to take public responsibility for its content.

## Funding

MK has received funding through the Gilead LEGA-C NoCo Program [Grant number IN-US-334-4492, June 1st 2018] for the development of this investigator-initiated study.

## Conflict of Interest

This study received funding from the Gilead LEGA-C NoCo Program. The funder had the following involvement with the study: revision of the manuscript. MK reports grants for investigator-initiated studies from ViiV Healthcare, Merck, and Gilead; consulting fees from ViiV Healthcare, Merck, AbbVie and Gilead. She is supported by a Canada Research Chair, Tier 1. BL is the holder of a Canadian Institutes for Health Research, Strategy for Patient-Oriented Research Mentorship Chair in Innovative Clinical Trials for HIV Care and also supported by a career award, LE 250, from the Quebec's Ministry of Health for researchers in Family Medicine. BL reports grants for investigator-initiated studies from ViiV Healthcare, Merck, and Gilead; consulting fees from ViiV Healthcare, Merck, and Gilead. DO-P, AA, TE, and KE have no conflicts of interest to declare.

## Publisher's Note

All claims expressed in this article are solely those of the authors and do not necessarily represent those of their affiliated organizations, or those of the publisher, the editors and the reviewers. Any product that may be evaluated in this article, or claim that may be made by its manufacturer, is not guaranteed or endorsed by the publisher.
