# Supplementary material for: Interventions to Improve Uptake of Direct-Acting Antivirals for Hepatitis C Virus in Priority Populations: A Systematic Review
Source: Front Public Health. 2022 Jun 24;10:877585. doi: 10.3389/fpubh.2022.877585 (PMC9263261; doi:10.3389/fpubh.2022.877585)
Supplement: Supplementary file 1 [file Data_Sheet_1.pdf]

Supplementary Material

1 Supplementary data

Supplementary Data 1. Search Strategies

All searches were initially run and results were exported on December 5, 2019. All searches were rerun on February 10, 2021 and results that were added or published between December 2019 and February 2021 were exported. The strategies and numbers below correspond to the last run of the search.

<u>BIOSIS (Clarivate Analytics)</u>

#	Query	Results
# 23	#22<br><br>Indexes=BCI Timespan=2013-2021	360
# 22	#21 OR #17 OR #15 OR #10<br><br>Indexes=BCI Timespan=All years	551
# 21	#20 AND #6	25
# 20	#19 OR #18	89,730
# 19	TS=((Australia* or northern territory or Tasmania or new south wales or Victoria or queensland) and (aborigin* or indigenous* or torres strait* islander*))	5,496
# 18	TS=(Athapaskan or Saulteaux or Wakashan or Cree or Dene or Inuit or Inuk or In uvialuit* or Haida or Ktunaxa or Tsimshian or Gitsxan or Nisga'a or Haisla or Hei ltsuk or Oweenkeno or Kwakwaka'wakw or Nuu chah nulth or Tsilhqot'in or Dake lh or Wet'suwet'en or Sekani or Dunne-<br>za or Dene or Tahltan or Kaska or Tagish or Tutchone or Nuxalk or Salish or Stl'at limc or Nlaka'pamux or Okanagan or Sec wepmc or Tlingit or Anishinaabe or Bla ckfoot or Nakoda or Tasttine or Tsuu T'inia or Gwich'in or Han or Tagish or Tutch one or Algonquin or Nipissing or Ojibwa or Potawatomi or Innu or Maliseet or Mi 'kmaq or Micmac or Passamaquoddy or Haudenosaunee or Cayuga or Mohawk or Oneida or Onodaga or Seneca or Tuscarora or Wyandot or Aboriginal* or Indigen ous* or Metis or red road or "on reserve" or off-<br>reserve or First Nation or First Nations or Amerindian or (urban NEAR/3 (Indian* or Native* or Aboriginal*)) or autochtone* or (Native* NEAR/1 (man* or men* or women* or woman* or boy* or girl* or adolescent* or youth or youths or	89,047

#	Query	Results
	person* or adult* or people* or Indian* or Nation or tribe* or tribal or band or bands)))	
# 17	#16 AND #6	67
# 16	TS=((men NEAR/2 sex* NEAR/4 men) or MSM or "sex between men" or ((gay or bisexual or homosexual) NEAR/2 (man or men)))	18,646
# 15	#6 and #14	471
# 14	#11 OR #12 or #13	40,087
# 13	TS=((medication-assisted NEAR/3 treatment) or ((opioid* or opiate*) NEAR/4 (replace* or substitut*)) or methadone* or buprenorphine* or naltrexone*)	24,557
# 12	TS=(PWID or "people who inject drugs")	1,338
# 11	TS=((intravenous* or parenteral* or inject* or IV) NEAR/3 (drug* or substance*) NEAR/8 (abuse or addict* or use* or using or people or person*))	16,655
# 10	#6 AND #9	44
# 9	#7 or #8	757,328
# 8	TS=(pregnant* or pregnanc* or prenatal* or pre-natal*)	434,964
# 7	TI=(woman* or women* or female* or girl* or sister* or mother* or daughter* or maternal*)	401,979
# 6	#1 AND #5	3,683

#	Query	Results
# 5	#2 or #3 or #4	142,625
# 4	TS=((treat* or therap*) NEAR/5 (uptake* or start* or initiat* or begin* or access* or link* or referr*))	142,104
# 3	TS=((Boceprevir or bocepravir or victrelis or Glecaprevir or Grazoprevir or Parita previr or veruprevir or Simeprevir or olysio or Voxilaprevir or Daclatasvir or dakl atasavir or daklatasvir or daklinza or dataclasvir or Elbasvir or Ledipasvir or Ombi tasvir or Pibrentasvir or Velpatasvir or Sofosbuvir or sovaldi or Dasabuvir or exvi era) and (uptake* or start* or initiat* or begin* or access* or link* or referr*))	779
# 2	TS=((DAA or DAAs or (direct* NEAR/2 (antiviral* or anti-viral*))) NEAR/5 (uptake* or start* or initiat* or begin* or access* or link* or referr*))	411
# 1	TS=(hepatitis-c or hep-c or HCV or ((non-a non-b) NEAR/3 hepatitis))<br><br>Indexes=BCI Timespan=All years	108,816

CINAHL (Ebsco)

#	Query	Results
S22	S21 Limiters - Published Date: 20130101-20201231	301
S21	S10 OR S14 OR S16 OR S20	404
S20	S6 and S19	27
S19	S17 or S18	33,705
S18	TI (((Australia* or northern territory or Tasmania or new south wales or Victoria or queensland) and (aborigin* or indigenous* or torres strait* islander*))) OR AB (((Australia* or northern territory or Tasmania or new south wales or Victoria or queensland) and (aborigin* or indigenous* or torres strait* islander*)))	4,790
S17	TI ((Athapaskan or Saulteaux or Wakashan or Cree or Dene or Inuit or Inuk or Inuvialuit* or Haida or Ktunaxa or Tsimshian or Gitsxan or Nisga'a or Haisla or Heiltsuk or Oweenkeno or Kwakwaka'wakw or Nuu chah nulth or Tsilhqot'in or	33,677

#	Query	Results
	Dakelh or Wet'suwet'en or Sekani or Dunne-za or Dene or Tahltan or Kaska or Tagish or Tutchone or Nuxalk or Salish or Stl'atlimc or Nlaka'pamux or Okanagan or Sec wepmc or Tlingit or Anishinaabe or Blackfoot or Nakoda or Tasttine or Tsuu T'inia or Gwich'in or Han or Tagish or Tutchone or Algonquin or Nipissing or Ojibwa or Potawatomi or Innu or Maliseet or Mi'kmaq or Micmac or Passamaquoddy or Haudenosaunee or Cayuga or Mohawk or Oneida or Onodaga or Seneca or Tuscarora or Wyandot or Aboriginal* or Indigenous* or Metis or red road or "on reserve" or off-reserve or First Nation or First Nations or Amerindian or (urban N3 (Indian* or Native* or Aboriginal*)) or autochtone* or (Native* N1 (man* or men* or women* or woman* or boy* or girl* or adolescent* or youth or youths or person* or adult* or people* or Indian* or Nation or tribe* or tribal or band or bands)))) OR AB ((Athapaskan or Saulteaux or Wakashan or Cree or Dene or Inuit or Inuk or Inuvialuit* or Haida or Ktunaxa or Tsimshian or Gitsxan or Nisga'a or Haisla or Heiltsuk or Oweenkeno or Kwakwaka'wakw or Nuu chah nulth or Tsilhqot'in or Dakelh or Wet'suwet'en or Sekani or Dunne-za or Dene or Tahltan or Kaska or Tagish or Tutchone or Nuxalk or Salish or Stl'atlimc or Nlaka'pamux or Okanagan or Sec wepmc or Tlingit or Anishinaabe or Blackfoot or Nakoda or Tasttine or Tsuu T'inia or Gwich'in or Han or Tagish or Tutchone or Algonquin or Nipissing or Ojibwa or Potawatomi or Innu or Maliseet or Mi'kmaq or Micmac or Passamaquoddy or Haudenosaunee or Cayuga or Mohawk or Oneida or Onodaga or Seneca or Tuscarora or Wyandot or Aboriginal* or Indigenous* or Metis or red road or "on reserve" or off-reserve or First Nation or First Nations or Amerindian or (urban N3 (Indian* or Native* or Aboriginal*)) or autochtone* or (Native* N1 (man* or men* or women* or woman* or boy* or girl* or adolescent* or youth or youths or person* or adult* or people* or Indian* or Nation or tribe* or tribal or band or bands))))	
S16	S6 AND S15	39
S15	TI (((men N2 sex* N4 men) or MSM or "sex between men" or ((gay or bisexual or homosexual) N2 (man or men)))) OR AB (((men N2 sex* N4 men) or MSM or "sex between men" or ((gay or bisexual or homosexual) N2 (man or men))))	14,485
S14	S6 and S13	351
S13	S11 OR S12	17,347
S12	TI ((PWID or "people who inject drugs")) OR AB ((PWID or "people who inject drugs")) OR TI (((medication-assisted NEAR/3 treatment) or ((opioid* or opiate*) NEAR/4 (replace* or substitut*)) or methadone* or buprenorphine* or naltrexone*)) OR AB (((medication-assisted NEAR/3 treatment) or ((opioid* or opiate*) NEAR/4 (replace* or substitut*)) or methadone* or buprenorphine* or naltrexone*))	10,977

#	Query	Results
S11	TI (((intravenous* or parenteral* or inject* or IV) N3 (drug* or substance*) N8 (abuse or addict* or use* or using or people or person*))) OR AB (((intravenous* or parenteral* or inject* or IV) N3 (drug* or substance*) N8 (abuse or addict* or use* or using or people or person*)))	8,469
S10	S6 AND S9	28
S9	S7 or S8	364,235
S8	TI ((pregnant* or pregnanc* or prenatal* or pre-natal*)) OR AB ((pregnant* or pregnanc* or prenatal* or pre-natal*))	159,321
S7	TI (woman* or women* or female* or girl* or sister* or mother* or daughter* or maternal*)	251,515
S6	S1 AND S5	1,315
S5	S2 or S3 or S4	63,543
S4	TI (((treat* or therap*) N5 (uptake* or start* or initiat* or begin* or access* or link* or referr*))) OR AB (((treat* or therap*) N5 (uptake* or start* or initiat* or begin* or access* or link* or referr*)))	63,432
S3	TI (((Boceprevir or bocepravir or victrelis or Glecaprevir or Grazoprevir or Paritaprevir or veruprevir or Simeprevir or olysio or Voxilaprevir or Daclatasvir or daklatasavir or daklatasvir or daklinza or dataclasvir or Elbasvir or Ledipasvir or Ombitasvir or Pibrentasvir or Velpatasvir or Sofosbuvir or sovaldi or Dasabuvir or exviera) and (uptake* or start* or initiat* or begin* or access* or link* or referr*))) OR AB (((Boceprevir or bocepravir or victrelis or Glecaprevir or Grazoprevir or Paritaprevir or veruprevir or Simeprevir or olysio or Voxilaprevir or Daclatasvir or daklatasavir or daklatasvir or daklinza or dataclasvir or Elbasvir or Ledipasvir or Ombitasvir or Pibrentasvir or Velpatasvir or Sofosbuvir or sovaldi or Dasabuvir or exviera) and (uptake* or start* or initiat* or begin* or access* or link* or referr*)))	189
S2	TI (((DAA or DAAs or (direct* N2 (antiviral* or anti-viral*))) N5 (uptake* or start* or initiat* or begin* or access* or link* or referr*))) OR AB (((DAA or DAAs or (direct* N2 (antiviral* or anti-viral*))) N5 (uptake* or start* or initiat* or begin* or access* or link* or referr*)))	202
S1	TI ((hepatitis-c or hep-c or HCV or ((non-a and non-b) N3 hepatitis))) OR AB ((hepatitis-c or hep-c or HCV or ((non-a and non-b) N3 hepatitis)))	16,806

Cochrane CENTRAL Register of Controlled Trials (Wiley)

ID	Query	Results
#1	(hepatitis-c or hep-c or HCV or ((non-a and non-b) NEAR/3 hepatitis)):ti,ab,kw	9965
#2	((DAA or DAAs or (direct* NEAR/2 (antiviral* or anti-viral*))) NEAR/5 (uptake* or start* or initiat* or begin* or access* or link* or referr*)):ti,ab,kw	64
#3	((Boceprevir or bocepravir or victrelis or Glecaprevir or Grazoprevir or Paritaprevir or veruprevir or Simeprevir or olysio or Voxilaprevir or Daclatasvir or daklatasavir or daklatasvir or daklinza or dataclasvir or Elbasvir or Ledipasvir or Ombitasvir or Pibrentasvir or Velpatasvir or Sofosbuvir or sovaldi or Dasabuvir or exviera) and (uptake* or start* or initiat* or begin* or access* or link* or referr*)):ti,ab,kw	275
#4	((treat* or therap*) NEAR/5 (uptake* or start* or initiat* or begin* or access* or link* or referr*)):ti,ab,kw	48954
#5	#2 or #3 or #4	49067
#6	#1 AND #5	862
#7	(woman* or women* or female* or girl* or sister* or mother* or daughter* or maternal*):ti,kw	770962
#8	(pregnant* or pregnanc* or prenatal* or pre-natal*):ti,ab,kw	69393
#9	#7 or #8	795275
#10	#6 AND #9	453
#11	((intravenous* or parenteral* or inject* or IV) NEAR/3 (drug* or substance*) NEAR/8 (abuse or addict* or use* or using or people or person*)):ti,ab,kw	1757
#12	(PWID or "people who inject drugs"):ti,ab,kw	219
#13	((medication-assisted NEAR/3 treatment) or ((opioid* or opiate*) NEAR/4 (replace* or substitut*)) or methadone* or buprenorphine* or naltrexone*):ti,ab,kw	7453
#14	#11 OR #12 or #13	8869

ID	Query	Results
#15	#6 and #14	119
#16	((men NEAR/2 sex* NEAR/4 men) or MSM or "sex between men" or ((gay or bisexual or homosexual) NEAR/2 (man or men))):ti,ab,kw	2209
#17	#6 AND #16	8
#18	(Athapaskan or Saulteaux or Wakashan or Cree or Dene or Inuit or Inuk or Inuvialuit* or Haida or Ktunaxa or Tsimshian or Gitsxan or Nisga'a or Haisla or Heiltsuk or Oweenkeno or Kwakwaka'wakw or Nuu chah nulth or Tsilhqot'in or Dakelh or Wet'suwet'en or Sekani or Dunne-za or Dene or Tahltan or Kaska or Tagish or Tutchone or Nuxalk or Salish or Stl'atlimc or Nlaka'pamux or Okanagan or Sec wepmc or Tlingit or Anishinaabe or Blackfoot or Nakoda or Tasttine or Tsuu T'inia or Gwich'in or Han or Tagish or Tutchone or Algonquin or Nipissing or Ojibwa or Potawatomi or Innu or Maliseet or Mi'kmaq or Micmac or Passamaquoddy or Haudenosaunee or Cayuga or Mohawk or Oneida or Onodaga or Seneca or Tuscarora or Wyandot or Aboriginal* or Indigenous* or Metis or red road or "on reserve" or off-reserve or First Nation or First Nations or Amerindian or (urban NEAR/3 (Indian* or Native* or Aboriginal*)) or autochtone* or (Native* NEAR/1 (man* or men* or women* or woman* or boy* or girl* or adolescent* or youth or youths or person* or adult* or people* or Indian* or Nation or tribe* or tribal or band or bands))):ti,ab,kw	10574
#19	((Australia* or northern territory or Tasmania or new south wales or Victoria or queensland) and (aborigin* or indigenous* or torres strait* islander*)):ti,ab,kw	382
#20	#18 or #19	10580
#21	#6 and #20	7
#22	#10 or #15 or #17 or #21	512

Embase (Ovid)

#	Query	Results
1	exp hepatitis C/	121627
2	(hepatitis-c or hep-c or HCV or ((non-a and non-b) adj3 hepatitis)).tw,kw.	150374

#	Query	Results
3	1 or 2	174284
4	(antivirus agent/ or (boceprevir or dasabuvir or glecaprevir or paritaprevir or simeprevir or sofosbuvir or voxilaprevir or daclatasvir or elbasvir or grazoprevir or ledipasvir or ombitasvir or pibrentasvir or velpatasvir).hw.) and (uptake* or start* or initiat* or begin* or access* or link* or referr*).tw,kw.	16421
5	((DAA or DAAs or (direct* adj2 (antiviral* or anti-viral*))) adj5 (uptake* or start* or initiat* or begin* or access* or link* or referr*)).tw,kw.	1798
6	((Boceprevir or bocepravir or victrelis or Glecaprevir or Grazoprevir or Paritaprevir or veruprevir or Simeprevir or olysio or Voxilaprevir or Daclatasvir or daklatasavir or daklatasvir or daklinza or dataclasvir or Elbasvir or Ledipasvir or Ombitasvir or Pibrentasvir or Velpatasvir or Sofosbuvir or sovaldi or Dasabuvir or exviera) and (uptake* or start* or initiat* or begin* or access* or link* or referr*)).tw,kw.	3514
7	((treat* or therap*) adj5 (uptake* or start* or initiat* or begin* or access* or link* or referr*)).tw,kw.	375941
8	4 or 5 or 6 or 7	386108
9	3 and 8	14658
10	Female/ not (Female/ and Male/)	3025995
11	(woman* or women* or female* or girl* or sister* or mother* or daughter* or maternal*).ti,kw.	723170
12	(pregnant* or pregnanc* or prenatal* or pre-natal*).tw,kw.	747775
13	10 or 11 or 12	3544714
14	9 and 13	1003
15	substance abuse/	55121
16	injection drug user/	2718

#	Query	Results
17	opiate substitution treatment/ or (methadone or buprenorphine).hw. or naltrexone/	60564
18	((intravenous* or parenteral* or inject* or IV) adj3 (drug* or substance*) adj8 (abuse or addict* or use* or using or people or person*)).tw,kw.	32372
19	(PWID or "people who inject drugs").tw,kw.	4236
20	((medication-assisted adj3 treatment) or ((opioid* or opiate*) adj4 (replace* or substitut*)) or methadone* or buprenorphine* or naltrexone*).tw,kw.	37592
21	or/15-20	143020
22	9 and 21	2246
23	male homosexuality/	3819
24	men who have sex with men/	11782
25	((men adj2 sex* adj4 men) or MSM or "sex between men" or ((gay or bisexual or homosexual) adj2 m?n)).tw,kw.	36686
26	23 or 24 or 25	39636
27	9 and 26	347
28	exp indigenous people/ or indigenous health care/ or Athapaskan.mp. or Saulteaux.mp. or Wakashan.mp. or Cree.mp. or Dene.mp. or Inuit.mp. or Inuk.mp. or Inuvialuit*.mp. or Haida.mp. or Ktunaxa.mp. or Tsimshian.mp. or Gitsxan.mp. or Nisga'a.mp. or Haisla.mp. or Heiltsuk.mp. or Oweenkeno.mp. or Kwakwaka'wakw.mp. or Nuu chah nulth.mp. or Tsilhqot'in.mp. or Dakelh.mp. or Wet'suwet'en.mp. or Sekani.mp. or Dunne-za.mp. or Dene.mp. or Tahltan.mp. or Kaska.mp. or Tagish.mp. or Tutchone.mp. or Nuxalk.mp. or Salish.mp. or Stl'atlimc.mp. or Nlaka'pamux.mp. or Okanagan.mp. or Sec wepmc.mp. or Tlingit.mp. or Anishinaabe.mp. or Blackfoot.mp. or Nakoda.mp. or Tasttine.mp. or Tsuu T'inia.mp. or Gwich'in.mp. or Han.mp. or Tagish.mp. or Tutchone.mp. or Algonquin.mp. or Nipissing.mp. or Ojibwa.mp. or Potawatomi.mp. or Innu.mp. or Maliseet.mp. or Mi'kmaq.mp. or Micmac.mp. or Passamaquoddy.mp. or Haudenosaunee.mp. or Cayuga.mp. or Mohawk.mp. or Oneida.mp. or Onodaga.mp. or Seneca.mp. or Tuscarora.mp. or Wyandot.mp. or Aboriginal*.mp. or Indigenous*.mp. or Metis.mp. or red road.mp. or "on reserve".mp. or off-reserve.mp. or First Nation.mp. or First Nations.mp. or Amerindian.mp. or (urban adj3 (Indian* or Native* or Aboriginal*)).mp. or	103991

#	Query	Results
	autochtone*.mp. or (Native* adj1 (man* or men* or women* or woman* or boy* or girl* or adolescent* or youth or youths or person* or adult* or people* or Indian* or Nation or tribe* or tribal or band or bands)).mp.	
29	(Australia.hw. or australia*.ti,ab. or au.in. or australia*.in. or northern territory.ti,ab. or northern territory.in. or tasmania.ti,ab. or Tasmania.in. or new south wales.ti,ab. or new south wales.in. or victoria.ti,ab. or Victoria.in. or queensland.ti,ab. or queensland.in.) and (exp oceanic ancestry group/ or aborigin*.ti,ab. or indigenous.mp. or torres strait* islander*.ti,ab.)	14943
30	28 or 29	104781
31	9 and 30	172
32	14 or 22 or 27 or 31	3400
33	limit 32 to yr="2013 -Current"	2578
34	limit 33 to (conference abstract or conference paper or "conference review")	1511
35	33 not 34	1067
36	limit 34 to yr="2017 -Current"	942
37	35 or 36	2009

Global Health (Ovid)

#	Query	Results
1	(hepatitis-c or hep-c or HCV or ((non-a and non-b) adj3 hepatitis)).mp.	40187
2	((DAA or DAAs or (direct* adj2 (antiviral* or anti-viral*))) adj5 (uptake* or start* or initiat* or begin* or access* or link* or referr*)).mp.	353
3	((Boceprevir or bocepravir or victrelis or Glecaprevir or Grazoprevir or Paritaprevir or veruprevir or Simeprevir or olysio or Voxilaprevir or Daclatasvir or daklatasavir or daklatasvir or daklinza or dataclasvir or	485

#	Query	Results
	Elbasvir or Ledipasvir or Ombitasvir or Pibrentasvir or Velpatasvir or Sofosbuvir or sovaldi or Dasabuvir or exviera) and (uptake* or start* or initiat* or begin* or access* or link* or referr*)).mp.	
4	((treat* or therap*) adj5 (uptake* or start* or initiat* or begin* or access* or link* or referr*)).mp.	40313
5	2 or 3 or 4	40593
6	1 and 5	2966
7	(woman* or women* or female* or girl* or sister* or mother* or daughter* or maternal*).ti.	138322
8	(pregnant* or pregnanc* or prenatal* or pre-natal*).ti,ab.	113596
9	7 or 8	210802
10	6 and 9	55
11	((intravenous* or parenteral* or inject* or IV) adj3 (drug* or substance*) adj8 (abuse or addict* or use* or using or people or person*)).mp.	14844
12	(PWID or "people who inject drugs").mp.	1748
13	((medication-assisted adj3 treatment) or ((opioid* or opiate*) adj4 (replace* or substitut*)) or methadone* or buprenorphine* or naltrexone*).mp.	2844
14	11 or 12 or 13	16731
15	6 and 14	580
16	((men adj2 sex* adj4 men) or MSM or "sex between men" or ((gay or bisexual or homosexual) adj2 m?n)).mp.	15717
17	6 and 16	72

#	Query	Results
18	(Athapaskan or Saulteaux or Wakashan or Cree or Dene or Inuit or Inuk or Inuvialuit* or Haida or Ktunaxa or Tsimshian or Gitsxan or Nisga'a or Haisla or Heiltsuk or Oweenkeno or Kwakwaka'wakw or Nuu chah nulth or Tsilhqot'in or Dakelh or Wet'suwet'en or Sekani or Dunne-za or Dene or Tahltan or Kaska or Tagish or Tutchone or Nuxalk or Salish or Stl'atlimc or Nlaka'pamux or Okanagan or Sec wepmc or Tlingit or Anishinaabe or Blackfoot or Nakoda or Tasttine or Tsuu T'inia or Gwich'in or Han or Tagish or Tutchone or Algonquin or Nipissing or Ojibwa or Potawatomi or Innu or Maliseet or Mi'kmaq or Micmac or Passamaquoddy or Haudenosaunee or Cayuga or Mohawk or Oneida or Onodaga or Seneca or Tuscarora or Wyandot or Aboriginal* or Indigenous* or Metis or red road or "on reserve" or off-reserve or First Nation or First Nations or Amerindian or (urban adj3 (Indian* or Native* or Aboriginal*)) or autochtone* or (Native* adj1 (man* or men* or women* or woman* or boy* or girl* or adolescent* or youth or youths or person* or adult* or people* or Indian* or Nation or tribe* or tribal or band or bands))).mp.	46626
19	(Australia.hw. or australia*.ti,ab. or au.in. or australia*.in. or northern territory.ti,ab. or northern territory.in. or tasmania.ti,ab. or Tasmania.in. or new south wales.ti,ab. or new south wales.in. or victoria.ti,ab. or Victoria.in. or queensland.ti,ab. or queensland.in.) and (oceanic ancestry group.hw. or aborigin*.ti,ab. or indigenous.mp. or torres strait* islander*.ti,ab.)	5876
20	18 or 19	46757
21	6 and 20	35
22	10 or 15 or 17 or 21	672
23	limit 22 to yr="2013 -Current"	465

Global Index Medicus (WHO)

#	Query	Results
1	tw:((tw:(hepatitis-c OR hep-c OR hcv)) AND (tw:(boceprevir OR bocepravir OR victrelis OR glecaprevir OR grazoprevir OR paritaprevir OR veruprevir OR simeprevir OR olysio OR voxilaprevir OR daclatasvir OR daklatasavir OR	9

	daklatasvir OR daklinza OR dataclasvir OR elbasvir OR ledipasvir OR ombitasvir OR pibrentasvir OR velpatasvir OR sofosbuvir OR sovaldi OR dasabuvir OR exviera OR daa OR daas OR direct-act* antiviral* OR direct-act* anti-viral*)) AND (tw:(uptake* OR start* OR initiat* OR begin* OR access* OR link* OR referr*)))	

<u>Medline (Ovid)</u>

#	Query	Results
1	exp Hepatitis C/	65170
2	(hepatitis-c or hep-c or HCV or ((non-a and non-b) adj3 hepatitis)).tw,kf.	92228
3	1 or 2	99830
4	(Antiviral Agents/ or simeprevir/ or sofosbuvir/) and (uptake* or start* or initiat* or begin* or access* or link* or referr*).tw,kf.	11733
5	((DAA or DAAs or (direct* adj2 (antiviral* or anti-viral*))) adj5 (uptake* or start* or initiat* or begin* or access* or link* or referr*)).tw,kf.	651
6	((Boceprevir or bocepravir or victrelis or Glecaprevir or Grazoprevir or Paritaprevir or veruprevir or Simeprevir or olysio or Voxilaprevir or Daclatasvir or daklatasavir or daklatasvir or daklinza or dataclasvir or Elbasvir or Ledipasvir or Ombitasvir or Pibrentasvir or Velpatasvir or Sofosbuvir or sovaldi or Dasabuvir or exviera) and (uptake* or start* or initiat* or begin* or access* or link* or referr*)).tw,kf.	951
7	((treat* or therap*) adj5 (uptake* or start* or initiat* or begin* or access* or link* or referr*)).tw,kf.	217787
8	4 or 5 or 6 or 7	225508
9	3 and 8	6817
10	Female/ not (Female/ and Male/)	2948524
11	(woman* or women* or female* or girl* or sister* or mother* or daughter* or maternal*).ti,kf.	604231

#	Query	Results
12	(pregnant* or pregnanc* or prenatal* or pre-natal*).tw,kf.	596036
13	10 or 11 or 12	3324162
14	9 and 13	326
15	Substance Abuse, Intravenous/	15432
16	Drug Users/	3282
17	Opiate Substitution Treatment/ or Methadone/ or exp Buprenorphine/ or Naltrexone/	25018
18	((intravenous* or parenteral* or inject* or IV) adj3 (drug* or substance*) adj8 (abuse or addict* or use* or using or people or person*)).tw,kf.	24194
19	(PWID or "people who inject drugs").tw,kf.	2964
20	((medication-assisted adj3 treatment) or ((opioid* or opiate*) adj4 (replace* or substitut*)) or methadone* or buprenorphine* or naltrexone*).tw,kf.	27252
21	or/15-20	62663
22	9 and 21	995
23	Homosexuality, Male/	16358
24	"Sexual and Gender Minorities"/ and Male/	3548
25	((men adj2 sex* adj4 men) or MSM or "sex between men" or ((gay or bisexual or homosexual) adj2 m?n)).tw,kf.	27461
26	23 or 24 or 25	33744
27	9 and 26	120
28	exp American Native Continental Ancestry Group/ or exp Health Services, Indigenous/ or exp Ethnopharmacology/ or Athapaskan.mp. or Saulteaux.mp. or Wakashan.mp. or Cree.mp. or Dene.mp. or Inuit.mp. or Inuk.mp. or Inuvialuit*.mp. or Haida.mp. or Ktunaxa.mp. or Tsimshian.mp. or Gitsxan.mp. or	88661

#	Query	Results
	Nisga'a.mp. or Haisla.mp. or Heiltsuk.mp. or Oweenkeno.mp. or Kwakwaka'wakw.mp. or Nuu chah nulth.mp. or Tsilhqot'in.mp. or Dakelh.mp. or Wet'suwet'en.mp. or Sekani.mp. or Dunne-za.mp. or Dene.mp. or Tahltan.mp. or Kaska.mp. or Tagish.mp. or Tutchone.mp. or Nuxalk.mp. or Salish.mp. or Stl'atlimc.mp. or Nlaka'pamux.mp. or Okanagan.mp. or Sec wepmc.mp. or Tlingit.mp. or Anishinaabe.mp. or Blackfoot.mp. or Nakoda.mp. or Tasttine.mp. or Tsuu T'inia.mp. or Gwich'in.mp. or Han.mp. or Tagish.mp. or Tutchone.mp. or Algonquin.mp. or Nipissing.mp. or Ojibwa.mp. or Potawatomi.mp. or Innu.mp. or Maliseet.mp. or Mi'kmaq.mp. or Micmac.mp. or Passamaquoddy.mp. or Haudenosaunee.mp. or Cayuga.mp. or Mohawk.mp. or Oneida.mp. or Onodaga.mp. or Seneca.mp. or Tuscarora.mp. or Wyandot.mp. or Aboriginal*.mp. or Indigenous*.mp. or Metis.mp. or red road.mp. or "on reserve".mp. or off-reserve.mp. or First Nation.mp. or First Nations.mp. or Amerindian.mp. or (urban adj3 (Indian* or Native* or Aboriginal*)).mp. or autochtone*.mp. or (Native* adj1 (man* or men* or women* or woman* or boy* or girl* or adolescent* or youth or youths or person* or adult* or people* or Indian* or Nation or tribe* or tribal or band or bands)).mp.	
29	(Australia.hw. or australia*.ti,ab. or au.in. or australia*.in. or northern territory.ti,ab. or northern territory.in. or tasmania.ti,ab. or Tasmania.in. or new south wales.ti,ab. or new south wales.in. or victoria.ti,ab. or Victoria.in. or queensland.ti,ab. or queensland.in.) and (oceanic ancestry group.hw. or aborigin*.ti,ab. or indigenous.mp. or torres strait* islander*.ti,ab.)	12283
30	28 or 29	90101
31	9 and 30	59
32	14 or 22 or 27 or 31	1381
33	limit 32 to yr="2013 -Current"	870

Scopus (Elsevier)

#	Query	Results
1	((((TITLE-ABS-KEY (hepatitis-c OR hep-c OR hcv OR ("non-a,non-b" W/3 hepatitis))) AND ((TITLE-ABS-KEY (((daa OR daas OR (direct* W/2 (antiviral* OR anti-viral*))) W/5 (uptake* OR start* OR initiat* OR begin* OR access* OR link* OR referr*)))) OR (TITLE-ABS-KEY (((boceprevir OR bocepravir OR victrelis OR glecaprevir OR grazoprevir OR paritaprevir OR veruprevir OR simeprevir OR olysio OR voxilaprevir OR daclatasvir OR	899

#	Query	Results
	daklatasavir OR daklatasvir OR daklinza OR dataclasvir OR elbasvir OR ledipasvir OR ombitasvir OR pibrentasvir OR velpatasvir OR sofosbuvir OR sovaldi OR dasabuvir OR exviera) AND (uptake* OR start* OR initiat* OR begin* OR access* OR link* OR referr*)))) OR (TITLE-ABS-KEY (((treat* OR therap*) W/5 (uptake* OR start* OR initiat* OR begin* OR access* OR link* OR referr*)))))) AND ((TITLE ((woman* OR women* OR female* OR girl* OR sister* OR mother* OR daughter* OR maternal*))) OR (TITLE-ABS-KEY ((pregnant* OR pregnanc* OR prenatal* OR pre-natal*))))) OR ((TITLE-ABS-KEY (((intravenous* OR parenteral* OR inject* OR iv) W/3 (drug* OR substance*) W/8 (abuse OR addict* OR use* OR using OR people OR person*)) OR (pwid OR "people who inject drugs" OR (medication-assisted W/3 treatment) OR ((opioid* OR opiate*) W/4 (replace* OR substitut*)) OR methadone* OR buprenorphine* OR naltrexone*))) AND ((TITLE-ABS-KEY (hepatitis-c OR hep-c OR hcv OR ("non-a,non-b" W/3 hepatitis))) AND ((TITLE-ABS-KEY (((daa OR daas OR (direct* W/2 (antiviral* OR anti-viral*))) W/5 (uptake* OR start* OR initiat* OR begin* OR access* OR link* OR referr*)))) OR (TITLE-ABS-KEY (((boceprevir OR bocepravir OR victrelis OR glecaprevir OR grazoprevir OR paritaprevir OR veruprevir OR simeprevir OR olysio OR voxilaprevir OR daclatasvir OR daklatasavir OR daklatasvir OR daklinza OR dataclasvir OR elbasvir OR ledipasvir OR ombitasvir OR pibrentasvir OR velpatasvir OR sofosbuvir OR sovaldi OR dasabuvir OR exviera) AND (uptake* OR start* OR initiat* OR begin* OR access* OR link* OR referr*)))) OR (TITLE-ABS-KEY (((treat* OR therap*) W/5 (uptake* OR start* OR initiat* OR begin* OR access* OR link* OR referr*))))))) OR (((TITLE-ABS-KEY (hepatitis-c OR hep-c OR hcv OR ("non-a,non-b" W/3 hepatitis))) AND ((TITLE-ABS-KEY (((daa OR daas OR (direct* W/2 (antiviral* OR anti-viral*))) W/5 (uptake* OR start* OR initiat* OR begin* OR access* OR link* OR referr*)))) OR (TITLE-ABS-KEY (((boceprevir OR bocepravir OR victrelis OR glecaprevir OR grazoprevir OR paritaprevir OR veruprevir OR simeprevir OR olysio OR voxilaprevir OR daclatasvir OR daklatasavir OR daklatasvir OR daklinza OR dataclasvir OR elbasvir OR ledipasvir OR ombitasvir OR pibrentasvir OR velpatasvir OR sofosbuvir OR sovaldi OR dasabuvir OR exviera) AND (uptake* OR start* OR initiat* OR begin* OR access* OR link* OR referr*)))) OR (TITLE-ABS-KEY (((treat* OR therap*) W/5 (uptake* OR start* OR initiat* OR begin* OR access* OR link* OR referr*)))))) AND (TITLE-ABS-KEY (((men W/2 sex* W/4 men) OR msm OR "sex between men" OR ((gay OR bisexual OR homosexual) W/2 (man OR men)))))) OR (((TITLE-ABS-KEY (hepatitis-c OR hep-c OR hcv OR ("non-a,non-b" W/3 hepatitis))) AND ((TITLE-ABS-KEY (((daa OR daas OR (direct* W/2 (antiviral* OR anti-viral*))) W/5 (uptake* OR start* OR initiat* OR begin* OR access* OR link* OR referr*)))) OR (TITLE-ABS-KEY (((boceprevir OR bocepravir OR victrelis OR glecaprevir OR grazoprevir OR paritaprevir OR veruprevir OR simeprevir OR olysio OR voxilaprevir OR daclatasvir OR daklatasavir OR daklatasvir OR daklinza OR dataclasvir OR elbasvir OR ledipasvir OR ombitasvir OR pibrentasvir OR velpatasvir OR sofosbuvir OR sovaldi OR dasabuvir OR exviera) AND (uptake* OR start* OR initiat* OR begin* OR access* OR link* OR referr*)))) OR (TITLE-ABS-KEY (((treat* OR therap*) W/5 (uptake* OR start* OR initiat* OR begin* OR access* OR link* OR referr*)))))) AND (TITLE-ABS-KEY ((athapaskan OR saulteaux OR wakashan OR	

#	Query	Results
	cree OR dene OR inuit OR inuk OR inuvialuit* OR haida OR ktunaxa OR tsimshian OR gitsxan OR nisga'a OR haisla OR heiltsuk OR oweenkeno OR kwakwaka'wakw OR nuu AND chah AND nulth OR tsilhqot'in OR dakelh OR wet'suwet'en OR sekani OR dunne-za OR dene OR tahltan OR kaska OR tagish OR tutchone OR nuxalk OR salish OR stl'atlimc OR nlaka'pamux OR okanagan OR sec AND wepmc OR tlingit OR anishinaabe OR blackfoot OR nakoda OR tasttine OR tsuu AND t'inia OR gwich'in OR han OR tagish OR tutchone OR algonquin OR nipissing OR ojibwa OR potawatomi OR innu OR maliseet OR mi'kmaq OR micmac OR passamaquoddy OR haudenosaunee OR cayuga OR mohawk OR oneida OR onodaga OR seneca OR tuscarora OR wyandot OR aboriginal* OR indigenous* OR metis OR red AND road OR "on reserve" OR off-reserve OR first AND nation OR first AND nations OR amerindian OR (urban W/3 (indian* OR native* OR aboriginal*)) OR autochtone* OR (native* W/1 (man* OR men* OR women* OR woman* OR boy* OR girl* OR adolescent* OR youth OR youths OR person* OR adult* OR people* OR indian* OR nation OR tribe* OR tribal OR band OR bands))) OR ((australia* OR northern AND territory OR tasmania OR new AND south AND wales OR victoria OR queensland) AND (aborigin* OR indigenous* OR torres AND strait* AND islander*)))))) AND (PUBYEAR > 2012)	

<u>Registries</u>

Source	Search Details	Results
ClinicalTrials.gov	In Expert Search:<br><br>AREA[ConditionSearch] Hepatitis C AND AREA[OutcomeSearch] (uptake OR access)	49
ICTRP	Initial Search:<br><br>In Basic Search:<br><br>1) Hepatitis C AND uptake = 10<br>2) Hepatitis C AND access = 66<br>Very slow to respond/access, due to COVID-19. Could not update.	1) 10<br>2) 66

Supplementary data 2. Sources of gray literature search

Databases

- Proquest Dissertations and Theses Global
    - ti((hepatitis-c OR hep-c OR HCV)) AND ((DAA OR DAAs OR (direct* NEAR/2 (antiviral* OR anti-viral*)) OR treat* OR therap*) NEAR/5 (uptake* OR start* OR initiat* OR begin* OR access* OR link* OR referr*))
    - Limit 2013-2020
    - 139 results
- PAIS Index
    - ti((hepatitis-c OR hep-c OR HCV)) AND ((DAA OR DAAs OR (direct* NEAR/2 (antiviral* OR anti-viral*)) OR treat* OR therap*) NEAR/5 (uptake* OR start* OR initiat* OR begin* OR access* OR link* OR referr*))
    - Limit 2013-2020
    - 130 results

Organizational Websites

- Canada Network of Hepatitis C: https://www.canhepc.ca/en
- CAHR: Canadian Association for HIV Research: https://www.cahr-acrv.ca/conference/archives/
- WHO—EU: http://www.euro.who.int/en/home
- Australian Society for HIV, Viral Hepatitis and Sexual Healthy: https://www.ashm.org.au/
- Hepatitis Australia: https://www.hepatitisaustralia.com/reports/
- CATIE: Canada's Source for HIV and Hepatitis C Information: https://www.catie.ca/en/home
- CIHR Canadian HIV Trials Network: http://www.hivnet.ubc.ca/

Governmental Websites

- Centres for Disease Control and Prevention, Division of Viral Hepatitis: https://www.cdc.gov/hepatitis/index.htm
- U.S Department of Health and Human Services| AIDSinfo: https://aidsinfo.nih.gov/
- Evidence in Health and Social Care: https://www.evidence.nhs.uk/

- Communicable Diseases and Infection Control| Public Health Agency of Canada: https://www.canada.ca/en/public-health/services/infectious-diseases.html

Conferences and Meetings

- Conference on Retroviruses and Opportunistic Infection: http://www.croiconference.org/
- International Symposium on Hepatitis Care in Substance Users: http://www.inhsu2018.com/ and http://www.inhsu2019.com/
- American Association for the Study of Liver Diseases: https://www.aasld.org/publications/hepatology-0
- The European Association for the Study of the Liver: http://www.easl.eu/
- International Aids Society: https://www.iasociety.org/
- Canadian Association for HIV Research: https://www.cahr-acrv.ca/

2 Supplementary Tables

Supplementary Table 1. Study characteristics

**Study**	**Type of full text**	**Research design**	**Country (Income)**	**DAA uptake intervention(s) described**	**Intervention description**	**Population targeted**	**DAA initiation among HCV+ participants**	**Critical appraisal (tool)**
Alavi et al.(31)	Manuscript	Non-randomized trial	Iran (Upper middle)	Colocation of care in community<br><br>Streamlined referral	OST clinics, community centres, homeless reception centres offering HCV testing and linkage to care at the POC, as well as DAA initiation for PWID. The program offers adherence support through directly observed dispensing of DAAs and count sheets.	632 PWID	Overall: 84%<br><br>- OST clinics: 100%<br><br>- Community centres: 96%<br><br>- Homeless centres: 54%	3/5 (MMAT)
Alimohammadi et al.(44)	Manuscript	Prospective cohort	Canada<br><br>(High)	Mobile clinic<br><br>Streamlined referral	A mobile clinic called "community pop-up clinic" offering POC simplified referrals to specialists and nurse care. Patients received rapid HCV testing and "a $10 gift card for completing testing and receiving the results" and a $10 meal voucher for scheduling an appointment.	435 Indigenous<br><br>942 PWID	61%	3/5 (MMAT)
Awali et al.(60)	Abstract	Prospective cohort	USA<br><br>(High)	Multidisciplinary team	A "comprehensive HCV treatment program employing a multidisciplinary team service." The team was integrated by infectious disease physicians, nurses and social workers.	190 PWID<br><br>35 MSM	51%	6/11 (STROBE)
Bielen et al.(41)	Abstract and conference poster	Prospective cohort	Belgium<br><br>(High)	Colocation of care in community<br><br>Care coordination<br><br>Patient education	A centre for drug addiction offering on-site HCV testing. HCV-positive patients are offered nurse-led case management, which involves referral to tertiary care, escort to medical appointment and "follow-up of compliance if treatment started." Patients are also educated on prevention of reinfection.	321 PWID	27% (2015; fibrosis needed >=F3)<br><br>31% (2017; fibrosis needed >=F2)	6/11 (STROBE)

Study	Type of full text	Research design	Country (Income)	DAA uptake intervention(s) described	Intervention description	Population targeted	DAA initiation among HCV+ participants	Critical appraisal (tool)
Bielen et al.(68)	Abstract	Prospective interventional cohort study	Belgium (High)	Colocation of care in community<br><br>Care coordination	An OST setting offering case management to provide support to PWID in "all the steps of the continuum of care."	334 PWID	43% (fibrosis needed >=F3)	5/11 (STROBE)
Buchanan and Ord(70)	Abstract	Prospective cohort	UK (High)	Colocation of care in community<br><br>Mobile clinic	"A HCV outreach treatment clinic" that was set alongside a needle exchange and harm reduction centre.	54 HCV+ PWID	77% (37/48 patients assessed for treatment)	5/11 (STROBE)
Chromy et al.(66)	Abstract	Prospective cohort	Austria (High)	Universal DAAs	Unrestricted DAA access	471 participants (58% PWID)	Intervention: 41%<br><br>Control 1 (Restricted DAAs): 32%<br><br>Control 2 (IFN): 27%	6/11 (STROBE)
Conti et al.(55)	Abstract	Prospective cohort	Italy (High)	Colocation of care in community<br><br>Patient education<br><br>Multidisciplinary team	A multidisciplinary team of addition physicians and hepatologist at an addition centre that offered HCV assessment and DAA initiation	165 PWID	98% (162/165)	6/11 (STROBE)
Cooper et al.(37)	Manuscript	Comparative cohort	Canada (High)	Telemedicine<br><br>Multidisciplinary team<br><br>Primary care setting	A telemedicine program that connects 1,583 clinics in Ontario with The Ottawa General Hospital. Nurses and patients in rural areas are connected with a multidisciplinary team of "HCV clinician, nurse and allied healthcare providers," who offer support.	110 PWID	Not previously linked: 20%<br><br>Control group (no intervention): 54%	5/5 (MMAT)

Study	Type of full text	Research design	Country (Income)	DAA uptake intervention(s) described	Intervention description	Population targeted	DAA initiation among HCV+ participants	Critical appraisal (tool)
Crouch et al.(42)	Abstract	Prospective cohort	USA (High)	Nurse-led care<br><br>Patient education<br><br>Peer-based intervention<br><br>Care coordination<br><br>Colocation of care in community	Nurse-led HCV care collocated in a syringe centre that offers patient education, group counselling, and case management.	11 PWID	11 participants	4/11 (STROBE)
Deriviere et al.(56)	Abstract	Prospective cohort	Australia (High)	Specialist supported General practitioner-led care<br><br>Provider education<br><br>Primary care setting	"Specialist-supported prescription of hepatitis C treatment by general practitioners." General practitioners are offered education. Specialists provide recommendations regarding HCV treatment and its monitoring.	134 HCV+ patients<br><br>32 Indigenous	76% (102/134)	5/11 (STROBE)
Doyle et al.(50)	Abstract	Prospective cohort	Australia (High)	Colocation of care in community<br><br>Mobile clinic<br><br>Nurse-led care<br><br>On-site DAA distribution	Mobile clinics "treating PWID and their injecting partners concurrently in community settings using a nurse-led model of care." Nurses performed assessments, FirboScans, HCV testing and delivered DAAs with the support of physicians.	114 PWID and 127 injecting partners	Primary PWID: 85%<br><br>Injecting partners: 55%	8/11 (STROBE)
Eckhardt et al.(45)	Manuscript	Prospective cohort	USA (High)	Colocation of care in community<br><br>On-site DAA distribution<br><br>Streamlined referrals	A needle exchange centre offering on-site HCV testing, care coordination, flexible appointments and drop-ins. DAAs were distributed on-site. Care coordination included education, adherence and social support.	80 PWID	66%	3/5 (MMAT)

Study	Type of full text	Research design	Country (Income)	DAA uptake intervention(s) described	Intervention description	Population targeted	DAA initiation among HCV+ participants	Critical appraisal (tool)
				Care coordination<br><br>Patient education				
Falade-Nwulia et al.(38)	Manuscript	Prospective cohort	USA (High)	Peer-based intervention<br><br>Patient education<br><br>Streamlined referrals	A "social network-based" intervention in which PWID with HCV are informed champions who "promote HCV testing and linkage to care within their injection drug networks." Champions provide education to members of their network, who were prompted to consult to an urban infectious disease clinic where they receive HCV rapid testing, linkage to care and DAAs.	100 PWID	Not previously linked: 20%<br><br>Previously linked: 34%	5/5 (MMAT)
Filippovych et al.(32)	Abstract	Prospective cohort	Ukraine (Lower-Middle)	Multidisciplinary team<br><br>Care coordination<br><br>Colocation of care in community	HCV care offered by multidisciplinary teams of doctors, nurses and case management implemented in "19 healthcare facilities (HCFs) in 16 regions of Ukraine in collaboration with 15 non-governmental organizations."	PWID (no information on total sample)	1203 participants (80% PWID; 79% HCV-HIV coinfected)	4/11 (STROBE)
Forns et al.(52)	Abstract	Comparative cohort	Spain (High)	Colocation of care in community<br><br>On-site DAA distribution	HCV testing, DAA initiation, and follow-ups were offered on-site at a harm reduction centres.	In harm reduction centres: 6878 PWID<br><br>In OST centres: 13944 PWID	Intervention: increased by 57% compared to SOC<br><br>In OST centres: increased by 19% compared to SOC	6/11 (STROBE)

**Study**	**Type of full text**	**Research design**	**Country (Income)**	**DAA uptake intervention(s) described**	**Intervention description**	**Population targeted**	**DAA initiation among HCV+ participants**	**Critical appraisal (tool)**
Freeman et al.(48)	Abstract	Prospective cohort	Australia (High)	Streamlined referral<br><br>Nurse-led care<br><br>Tertiary care setting	A nurse-led intervention consisting of the identification of patients at risk of HCV infection by screening hospital admissions and electronic medical records. "DAA treatment was initiated either while an inpatient or on discharge. Patients were offered follow-up appointments at a liver clinic and/or linkage to community-based care with general practitioners, support workers, and community nurses."	20 participants (85% PWID)	65%	6/11 (STROBE)
Gottfredsson et al.(62)	Abstract	Prospective cohort	Iceland (High)	DAA universal access	Universal access to DAAs defined as: "Starting in 01/2016 with the TraP HepC program, all HCV patients in Iceland have been offered DAAs."	557 participants (89% PWID)	94% (526/557 of HCV+ patients)	4/11 (STROBE)
Harney et al.(58)	Manuscript	Prospective cohort	Australia (High)	Nurse-led care<br><br>Colocation of care in community<br><br>Provider education<br><br>Multidisciplinary team	An HCV nurse-led service offered at two homeless facilities. Staff received "HCV education and training by a social worker and project coordinator to enhance identification and referral to the nurse service." Nurses visit the facilities on a weekly basis to engage patients and conduct HCV testing. Affiliated doctors provide DAA prescription, while nurses "attempted to retain clients in care until SVR blood test, including through phone calls and contact with health providers."	67 participants (43% PWID)	62% (24/39 HCVI+ participants)	4/5 (MMAT)

Study	Type of full text	Research design	Country (Income)	DAA uptake intervention(s) described	Intervention description	Population targeted	DAA initiation among HCV+ participants	Critical appraisal (tool)
Harrison et al.(35)	Manuscript	Non-randomized trial	UK (High)	Colocation of care in community<br><br>Provider education<br><br>Care coordination<br><br>Streamlined referrals<br><br>Peer support	Facilitators at drug dependence centres offering education to staff who have direct contact with PWID, simplified referral pathways and reminders using patient-specific means of communication. The intervention also comprised on-site DBS testing and the introduction of peer support programs with local champions.	4301 participants in intervention sites (38.9% PWID)<br><br>2338 participants in control sites (35% PWID)	Intervention sites: increased treatment initiation (OR=21.4, CI = 8.2–56.1), with difference of 13 % (CI = 9–16%) compared to baseline.<br><br>Control sites: little change in treatment initiation with -1% difference (CI=-6 to +3%; OR=0.74, CI=0.29-1.88)	5/5 (MMAT)
Howell et al.(47)	Abstract	Non-randomized trial	Australia (High)	Colocation of care in community<br><br>Streamlined referrals	Needle and syringe clinic offering mouth swab HCV screening and on-site serum viral load tests. Results were provided the same day of the tests. HCV positive patients received a follow-up appointment to link to care.	174 PWID	44% (31/70 of HCV + participants)	8/11 (STROBE)
Hsiang et al.(69)	Abstract	Cluster randomized trial	Singapore (High)	Secondary care setting	HCV positive patients were cluster randomized to two groups: standard access (i.e., primary care physician visit and referral to hospital specialist) versus “direct open access specialist clinic designed to treat community HCV patients.”	308 ex-PWID	Intervention: 38%<br><br>Control (SOC): 6.0%	8/11 (STROBE)
Martel-Laferriere et al.(49)	Abstract	Historical comparative cohort	Canada (High)	Accelerated model of care<br><br>On-site DAA distribution	Single-day evaluation and DAA initiation upon second visit.	99 PWID in intervention<br><br>76 PWID in historical cohort	Intervention: 79% (48/61), 70 days (52-106) between assessment and initiation.<br><br>Historical cohort: 66% (21/32), 266 days (150-449) between assessment and initiation.	9/11 (STROBE)

Study	Type of full text	Research design	Country (Income)	DAA uptake intervention(s) described	Intervention description	Population targeted	DAA initiation among HCV+ participants	Critical appraisal (tool)
Martinello et al.(65)	Manuscript	Multicentre cohort study	Australia (High)	DAA universal access	Universal access to DAAs defined as: "on March 1st 2016, DAA therapy was made available to all adults living with HCV via the Pharmaceutical Benefits Scheme"	402 participants (54% PWID; 32% MSM)	2014: 7% (95%CI 4%, 10%)<br><br>2015: 11% (95%CI 7%, 14%)<br><br>2016: 80% (95%CI 74%, 84%)<br><br>2017: 35% (95%CI 25%, 46%)	4/5 (MMAT)
Mazhnaya et al.(33)	Manuscript	Prospective cohort	Ukraine (Lower-Middle)	Colocation of care in community<br><br>Multidisciplinary team<br><br>Care coordination<br><br>Streamlined referrals<br><br>Patient education	"a community-involved treatment model with close collaboration between medical staff, representatives from NGOs and patients themselves." Social workers from the NGO are in charge of case management, counselling, scheduling of medical appointments, and promote adherence to HCV care by individualized reminders. Patients receive education on drug use and reinfection preventions.	1126 participants (80% PWID)	98%	4/5 (MMAT)
Mohsen et al.(57)	Manuscript	Retrospective cohort	Australia (High)	Multidisciplinary team<br><br>Provider education	Specialists and community doctors work together as a team. Collaboration is possible thanks to video conference meetings in which they share knowledge through case-based learning.	100 participants in intervention (32% PWID; 15% Indigenous)<br><br>100 participants in control (12% PWID; 5% Indigenous)	Intervention: 78%<br><br>Control (tertiary care): 81%	4/5 (MMAT)
Morris et al.(43)	Manuscript	Prospective cohort	Australia (High)	Case management<br><br>Needle exchange setting<br><br>Patient education	A community-based intervention at a needle exchange setting that incorporates case management, counselling, and peer education. Case managers (i.e., "harm reduction officers") are in charge of offering reminders for testing and taking medication.	476 participants (47% PWID: 12% Indigenous)	72% (342/476)	4/5 (MMAT)

Study	Type of full text	Research design	Country (Income)	DAA uptake intervention(s) described	Intervention description	Population targeted	DAA initiation among HCV+ participants	Critical appraisal (tool)
Norton et al.(46)	Abstract	Comparative cohort	USA (High)	Primary care setting<br><br>Patient education<br><br>Group medical visits<br><br>Streamlined referrals	Group visits at a primary care setting where physicians offered "HCV related medical evaluations, education, support, and skill building." Patients received an expedited appointment for DAA initiation at the primary care clinic.	40 PWID	57% (4/7 of enrolled participants)	8/11 (STROBE)
Puigvehi et al.(59)	Abstract	Prospective cohort	Spain (High)	Multidisciplinary team	Multidisciplinary team of primary care physicians, specialists, pharmacists, and specialists in hepatology, mental health and internal medicine. The team was in charge of screening, evaluation, treatment initiation and follow-up.	101 participants (19% PWID)	22% (22/101)	8/11 (STROBE)
Radley et al.(40)	Manuscript	Randomized controlled trial	UK (High)	Pharmacist-led care<br><br>On-site DAA distribution<br><br>Colocation of care in community	Community pharmacies offering OST and HCV testing, which are able to start treatment on the spot if there are no contraindications. Pharmacists may communicate with hepatologist should they have any queries.	341 PWID in intervention<br><br>338 PWID in control	Intervention: 33% (112/341) of HCV + participants<br><br>Control (SOC): 17% (61/338) of HCV + participants	2/5 (MMAT)
Saeed et al.(63)	Manuscript	Quasi-experimental trial	Canada (High)	DAA universal access	Universal access to DAAs defined as "the change in provincial policies that removed the criterion requiring the presence of a 'significant liver fibrosis stage' for DAAs to be reimbursed by 3 provincial health plans."	1130 participants (18% Indigenous; 27% MSM; 35% PWID)	Estimated to increase 1.8-fold (95% CI, 1.4–2.4)	4/5 (MMAT)
Talal et al.(53)	Manuscript	Prospective cohort	USA (High)	Telemedicine<br><br>On-site DAA distribution<br><br>Colocation of care in community	After having two HCV education sessions, patients at a OST centre were offered HCV testing. If HCV RNA+ they received consultation via telemedicine with a hepatologist. The hepatologist later had a discussion with an OST advanced practitioner to define the treatment plan. DAAs were later dispensed at the OST centre along with methadone.	62 PWID	73% (45/62) of HCV RNA+ participants	3/5 (MMAT)

**Study**	**Type of full text**	**Research design**	**Country (Income)**	**DAA uptake intervention(s) described**	**Intervention description**	**Population targeted**	**DAA initiation among HCV+ participants**	**Critical appraisal (tool)**
				Patient education<br><br>Multidisciplinary team				
Thomas et al.(61)	Abstract	Prospective cohort	Australia (High)	Specialist supported General practitioner-led care<br><br>Primary care setting	Metropolitan primary care doctors provide information of HCV positive cases to a hepatologist by fax or email who, in turn, provide advice, determine the need for more investigations and establish a plan for DAA therapy. "This was then returned to the treating doctor to prescribe DAA therapy without requiring routine hospital attendance, doctor to doctor teleconference or phone call or specialist tests."	257 participants<br><br>(43% PWID)	90% (230/257)	6/11 (STROBE)
Traeger et al.(64)	Manuscript	Retrospective cohort	Australia (High)	DAA universal access	DAA universal access defined as: "the decision to make DAA treatments available through the national Pharmaceutical Benefits Scheme (PBS) in 2016."	113,832 in intervention (9.7% PWID)<br><br>93,856 in control (11.2% PWID)	Intervention: 45% (1671/3713)<br><br>Control (historical): 1% (19/2515)	4/5 (MMAT)
Vasileiadi et al.(67)	Abstract	Historical comparative cohort	Greece (High)	DAA universal access	DAA access without restrictions (i.e., fibrosis or comorbidities).	757 participants (50% PWID)	Intervention: 29 patients treated/month<br><br>With restrictions: 15 patients treated/month	6/11 (STROBE)
Wade et al.(39)	Manuscript	Randomized controlled trial	Australia and New Zealand (High)	Primary care setting<br><br>Multidisciplinary team<br><br>Nurse-led care	Primary care teams of general practitioners offering OST; an infectious disease physician conducting a viral hepatitis clinic every 2 weeks; and nurses who see the patients worked them up, gave them the scripts and monitored their treatment.	70 PWID in intervention<br><br>66 PWID in control	Intervention: 75%, 43/57<br><br>Control (SOC): 34%, 18/53	3/5 (MMAT)

Study	Type of full text	Research design	Country (Income)	DAA uptake intervention(s) described	Intervention description	Population targeted	DAA initiation among HCV+ participants	Critical appraisal (tool)
							P < .001, RR=2.48 (95% CI 1.54–3.95)	
Ward et al.(34)<br><br>*Companion paper of Harrison et al.(35)*	Manuscript	Cost-effectivenes s study	UK (High)	Colocation of care in community<br><br>Provider education<br><br>Care coordination<br><br>Streamlined referrals<br><br>Peer support	Facilitators at drug dependence centres offering: education to staff who have direct contact with PWID, simplified referral pathways and reminders using patient-specific means of communication. The intervention also comprised on-site DBS testing and the introduction of peer support programs with local champions.	4301 participants in intervention sites (38.9% PWID)<br><br>2338 participants in control sites (35% PWID)	Estimated to increase 2.9-fold annually (95%CI 2.3-6.1).	NA
Wigglesworth et al.(54)	Abstract	Prospective cohort	UK (High)	Patient education<br><br>Colocation of care in community<br><br>On-site DAA distribution	Offering on-site pangenomic treatment to female workers that tested HCV RNA+ at community organization. They also received counselling	46 Female sex workers	13/18 HCV+ female sex workers	7/11 (STROBE)
Wungjiranirun et al.(51)	Abstract	Prospective cohort	USA (High)	Mobile clinic<br><br>Telemedicine<br><br>On-site DAA distribution	Mobile clinics offering HCV testing, fibroscan, pre-treatment laboratories, telemedicine consultation, DAA initiation, and follow-up.	190 PWID	16/32 of those that tested positive	5/11 (STROBE)

CI: confidence interval; DAA: direct-acting antivirals; HCV: hepatitis C virus; MSM: men who have sex with men; OR: odds ratio; OST: opioid substitution therapy; POC: point of care; PWID: people who inject drugs; RR: risk ratio; SOC: standard of care; SVR: sustained virologic response

Supplementary Table 2. Intervention categories in each included study

Study	Interventions targeting patients			Interventions targeting providers				Interventions targeting the Health System				
	Care coordination	Accelerated DAA initiation & on-site distribution	Patient education	Provider education	Telemedicine	Multidisciplinary teams	GP-led care	DAA universal access	Primary care setting	Secondary care setting	Tertiary care setting	Colocation in community settings
Alavi et al.(31)	X											X
Alimohammadi et al.(44)	X											X
Awali et al.(60)						X						
Bielen et al.(41)	X											X
Bielen et al.(68)	X											X
Buchanan and Ord(70)												X
Chromy et al.(66)								X				
Conti et al.(55)			X			X						X
Cooper et al.(37)					X	X			X			
Crouch et al.(42)	X		X			X						X
Deriviere et al.(56)				X			X		X			
Doyle et al.(50)		X				X						X
Eckhardt et al.(45)	X	X	X									X
Falade-Nwulia et al.(38)	X		X									
Filippovych et al.(32)	X					X						X
Forns et al.(52)		X										X

Study	Interventions targeting patients			Interventions targeting providers				Interventions targeting the Health System				
	Care coordination	Accelerated DAA initiation & on-site distribution	Patient education	Provider education	Telemedicine	Multidisciplinary teams	GP-led care	DAA universal access	Primary care setting	Secondary care setting	Tertiary care setting	Colocation in community settings
Freeman et al.(48)	X					X					X	
Gottfredsson et al.(62)								X				
Harney et al.(58)				X		X						X
Harrison et al.(35)	X		X	X								X
Howell et al.(47)	X											X
Hsiang et al.(69)										X		
Martel-Laferriere et al.(49)		X										
Martinello et al.(65)								X				
Mazhnaya et al.(33)	X		X			X						X
Mohsen et al.(57)				X		X						
Morris et al.(43)	X		X									X
Norton et al.(46)	X		X						X			
Puigvehi et al.(59)						X						
Radley et al.(40)		X				X						X
Saeed et al.(63)								X				
Talal et al.(53)		X	X		X	X						X

Study	Interventions targeting patients			Interventions targeting providers				Interventions targeting the Health System				
	Care coordination	Accelerated DAA initiation & on-site distribution	Patient education	Provider education	Telemedicine	Multidisciplinary teams	GP-led care	DAA universal access	Primary care setting	Secondary care setting	Tertiary care setting	Colocation in community settings
Thomas et al.(61)							X		X			
Traeger et al.(64)								X				
Vasileiadi et al.(67)								X				
Wade et al.(39)						X			X			
Ward et al.(34)	X		X	X								X
Wigglesworth et al.(54)		X	X									X
Wungjiranirun et al.(51)		X			X	X						X
TOTAL	15	8	11	5	3	15	2	6	5	1	1	21

DAA: direct-acting antiviral; GP: general practitioner
